# Molecular Detection of *Theileria ovis*, *Anaplasma ovis,* and *Rickettsia* spp. in *Rhipicephalus turanicus* and *Hyalomma anatolicum* Collected from Sheep in Southern Xinjiang, China

**DOI:** 10.3390/pathogens13080680

**Published:** 2024-08-11

**Authors:** Yongchang Li, Jianlong Li, Gulaimubaier Xieripu, Mohamed Abdo Rizk, Adrian Miki C. Macalanda, Lu Gan, Jichao Ren, Uday Kumar Mohanta, Shimaa Abd El-Salam El-Sayed, Bayin Chahan, Xuenan Xuan, Qingyong Guo

**Affiliations:** 1 Parasitology Laboratory, Veterinary College, Xinjiang Agricultural University, Urumqi 830011, China; yongchangli@xjau.edu.cn (Y.L.); pbb0108@163.com (J.L.); 18699191484@163.com (G.X.); 13079971281@163.com (L.G.); 18999574197@163.com (J.R.); bynch@hotmail.com (B.C.); 2 National Research Center for Protozoan Diseases, Obihiro University of Agriculture and Veterinary Medicine, Obihiro 080-8555, Japan; dr_moh_abdo2008@mans.edu.eg (M.A.R.); adrian_macalanda@yahoo.com (A.M.C.M.); uday_vet01@yahoo.com (U.K.M.); shimaa_a@mans.edu.eg (S.A.E.-S.E.-S.); 3 Department of Internal Medicine and Infectious Diseases, Faculty of Veterinary Medicine, Mansoura University, Mansoura 35516, Egypt; 4 Department of Immunopathology and Microbiology, College of Veterinary Medicine and Biomedical Sciences, Cavite State University, Indang 4122, Philippines; 5 Department of Microbiology and Parasitology, Sher–e–Bangla Agricultural University, Sher–e–Bangla Nagar, Dhaka 1207, Bangladesh; 6 Department of Biochemistry and Chemistry of Nutrition, Faculty of Veterinary Medicine, Mansoura University, Mansoura 35516, Egypt

**Keywords:** *Theileria ovis*, *Anaplasma ovis*, *Rickettsia*, *Hyalomma anatolicum*, *Rhipicephalus turanicus*, Xinjiang, China

## Abstract

The Xinjiang Uygur Autonomous Region (Xinjiang) borders eight countries and has a complex geographic environment. There are almost 45.696 million herded sheep in Xinjiang, which occupies 13.80% of China’s sheep farming industry. However, there is a scarcity of reports investigating the role of sheep or ticks in Xinjiang in transmitting tick-borne diseases (TBDs). A total of 894 ticks (298 tick pools) were collected from sheep in southern Xinjiang. Out of the 298 tick pools investigated in this study, *Rhipicephalus turanicus* (*Rh. turanicus*) and *Hyalomma anatolicum* (*H. anatolicum*) were identified through morphological and molecular sequencing. In the southern part of Xinjiang, 142 (47.65%), 86 (28.86%), and 60 (20.13%) tick pools were positive for *Rickettsia* spp., *Theileria* spp., and *Anaplasma* spp., respectively. Interestingly, the infection rate of *Rickettsia* spp. (73%, 35.10%, and 28.56–41.64%) was higher in *Rh. turanicus* pools than in *H. anatolicum* pools (4%, 4.44%, and 0.10–8.79%) in this study. Fifty-one tick pools were found to harbor two pathogens, while nineteen tick pools were detected to have the three pathogens. Our findings indicate the presence of *Rickettsia* spp., *Theileria* spp., and *Anaplasma* spp. potentially transmitted by *H. anatolicum* and *Rh. turanicus* in sheep in southern Xinjiang, China.

## 1. Introduction

Ticks and tick-borne diseases (TBDs) are a growing global problem that can have a detrimental impact on both human and animal health. As ticks are the second most common vectors worldwide, the biosurveillance of TBDs, such as piroplasmosis (babesiosis and theileriosis), anaplasmosis, and rickettsiosis, is crucial for protecting public and animal health. Ticks and TBDs negatively affect animal production, leading to pruritus, weight loss, reduced immunity, fever, anemia, and even death [1].

In Asia, tick-borne pathogens have become a concern in many countries in recent years, including China, Mongolia, India, Tajikistan, and Pakistan [2,3]. The identification and control of several tick-borne diseases are also a concern for sheep. Sheep can be affected by TBDs, such as *Theileria* spp., *Anaplasma* spp., and *Rickettsia* spp. Additionally, these pathogens were detected near the border of the Xinjiang Uygur Autonomous Region (Xinjiang). For example, the *Theileria ovis* (*T. ovis*), *Anaplasma ovis* (*A. ovis*), and *Rickettsia* species have been reported in Pakistan and Tajikistan [4]. 

Importantly, Xinjiang shares its borders with eight other countries and has a complex geographic environment that includes diverse features, such as mountains, plateaus, deserts, and basins. This region is typically divided into two distinct climatic zones: the north, with a temperate continental arid and semi-arid climate, and the south with a warm temperate continental arid climate. These two zones are separated by the Tianshan Mountains [5]. According to the Xinjiang Statistics Bureau (https://tjj.xinjiang.gov.cn/tjj/tjgn/202203/7ab304445f174a7eb1f5165be4f94041.shtml, accessed on 25 December 2023) [6], Xinjiang has approximately 45.696 million sheep, comprising 13.8% of China’s total sheep farming industry. However, despite the importance of the sheep industry in Xinjiang and China as a whole, there are few studies on ticks in sheep, which are known to transmit tick-borne diseases (TBDs) or tick-borne pathogens (TBPs).

A previous study identified 12 tick species on five animal species, including sheep, in northern Xinjiang counties [7]. However, information about the pathogens carried or transmitted by ticks in southern Xinjiang remains limited. Therefore, this study was conducted to identify and characterize the pathogens harbored or transmitted by tick species collected from sheep in southern Xinjiang.

## 2. Materials and Methods

### 2.1. Tick Sample Collection

**Figure 1:**
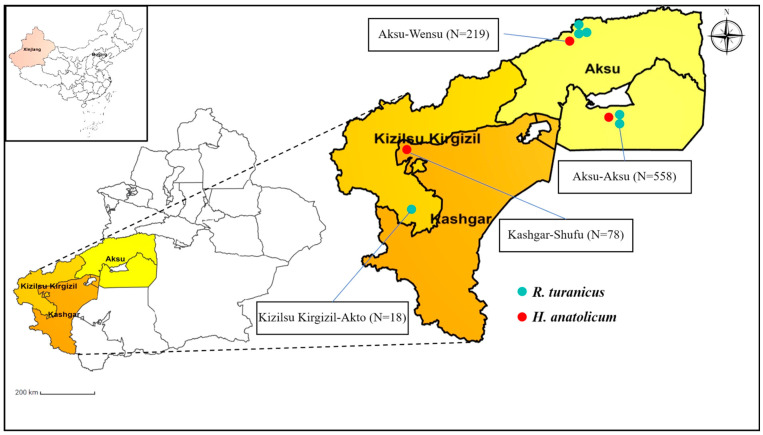
Map of Aksu (Aksu and Wensu), Kashgar (Shufu), and Kizilsu Kirgizil (Akto) of Xinjiang, China, with the sampling sites included in the study.

A total of 894 partially engorged adult ticks (female and male) were randomly collected from three districts (Aksu, Kashgar, and Kizilsu Kirgizil) and seven locations in southern Xinjiang, China. Farms A, B, and C are located in Aksu-Aksu, while Farms D and E are in Aksu-Wensu. Additionally, ticks were also collected from Kashgar-Shufu and Kizilsu Kirgizil-Akto (Figure 1). These ticks were collected from the body surfaces of 128 asymptomatic sheep across four regions: Aksu (48 sheep), Wensu (40 sheep), Shufu (20 sheep), and Akto County (20 sheep). Briefly, the ticks were collected from the surface of the sheep, including the ear, perineum, perianal areas, and the base of the tail [7]. The ticks were initially washed with phosphate-buffered saline (PBS, pH 7.2) and subsequently sonicated using ultrasonic waves (80 kHz) for 30 min. All ticks were immersed in boiling water and individually preserved in labeled vials containing 70% ethanol. Before DNA extraction, the ticks were again washed with 70% ethanol and air-dried. The protocol for this study was approved by the Committee on the Ethics of Animal Experiments at the Obihiro University of Agriculture and Veterinary Medicine, Japan (Permit numbers: animal experiment, 230244; DNA experiment, AP0001299621; Pathogen, AP0001299622; Ethical approval numbers, 22–23 and 23–17). 

### 2.2. Tick Identification

The collected ticks were identified morphologically using published identification keys [8,9]. Specifically, the genera of the ticks were classified based on features such as the basis capituli, scutum, and spiracular plates [8,9]. 

### 2.3. DNA Extraction

In each sampling county, tick specimens morphologically identified as the same tick species were randomly selected for pathogen detection. Three ticks from the same region and farm, morphologically identified as the same species, were pooled. This pool was washed three times with 1× PBS, ground in liquid nitrogen using a mortar and pestle, and resuspended in 200 μL of 1× PBS. Genomic DNA was extracted from this suspension using a resin-based DNA extraction kit (TIANGEN, Beijing, China) and stored at −20 °C according to the manufacturer’s instructions. 

### 2.4. Molecular Characterization of the Tick Species and Characterization of Tick-Borne Pathogens

The molecular identification of the most common tick species was carried out using PCR assays. Target primers for partial sequences of *Babesia* spp. 18S rRNA, *Theileria* spp. 18S rRNA, *Rickettsia* spp. outer membrane protein (OmpA), and *Anaplasma* spp. major surface protein (Msp4) were employed [7,10,11,12]. In brief, all adult ticks (894) were screened via PCR using the primers listed in Table A1. PCR products were visualized on a 1.0% agarose gel stained with ethidium bromide under UV light [7]. 

All amplicons were extracted from the gels using a TIANquick Midi Purification Kit (TIANGEN, Beijing, China) and sequenced. The purified PCR products (pathogen DNA amplicons) were cloned using a pGEM-T Easy Vector (Promega, Madison, WI, USA). The plasmids were sent to Shanghai Sangon Company for sequencing [7]. 

The identities and similarities of the sequenced isolates were assessed using the BLASTn tool against the NCBI GenBank database and the Clustal X program. Phylogenetic trees were constructed using the maximum likelihood (ML) method with the Kimura 2-parameter model in MEGA version 7.0. Bootstrap support values (1000 replicates) were indicated at each node. Confidence intervals (95% CI) and one-way ANOVA analyses were conducted using GraphPad Prism 6.0.

## 3. Results

### 3.1. Rh. turanicus and H. anatolicum Are the Identified Tick Species Based on Morphology and Molecular Methods

The morphological and molecular observation identified adult ticks (894) belonging to two tick genera, 624 (208 pools) to *Rhipicephalus turanicus* (*Rh. turanicus*) and 270 (90 pools) to *Hyalomma anatolicum* (*H. anatolicum*), which were collected from sheep (Figure 1; Table 1). 

### 3.2. Pathogens

Three pathogens were identified in the pools of *Rh. turanicus* and *H. anatolicum*. The total infection rates of *Rickettsia* spp., *Anaplasma* spp., and *Theileria* spp. were 47.65% (142/298), 20.13% (60/298), and 28.86% (86/298), respectively. Both ticks were investigated for *Rickettsia* spp. in Aksu and Wensu, Xinjiang (Table 1). Interestingly, the infection rate of *Rickettsia* spp. (35.10%, with a range of 28.56–41.64%) was higher and more easily identified in *Rh. turanicus* (73 samples) compared to *H. anatolicum* (4 samples, 4.44%, with a range of 0.10–8.79%). A total of 51 ticks had double infections, and 19 had triple infections. The most common co-infection was *A. ovis* + *Rickettsia* spp. (15.38%, 10.44–20.33), followed by *Rickettsia* spp. + *T. ovis* (4.70%, 2.28–7.11) and *A. ovis* + *T. ovis* (1.68%, 0.21–3.15) (Table 2). Nineteen *Rh. turanicus* pools (9.14%, 5.19–13.08) carried all three pathogens *Rickettsia* spp., *A. ovis*, and *T. ovis*, while only *Rickettsia* spp. and *T. ovis* were identified as co-infections in *H. anatolicum*.

### 3.3. Analyses of the Three Pathogens’ DNA Sequences

**Figure 2:**
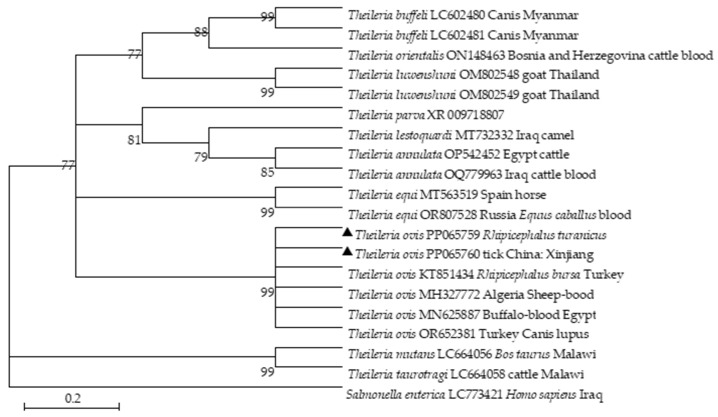
The maximum likelihood method based on the Kimura 2-parameter model was used to investigate *Theileria* spp. Phylogenetic analysis of the 18S rRNA gene sequence was performed. The number marked at each node represents the percentage occurring in 1000 bootstrap repetitions. The sequences in this study are marked with black triangles.

**Figure 3:**
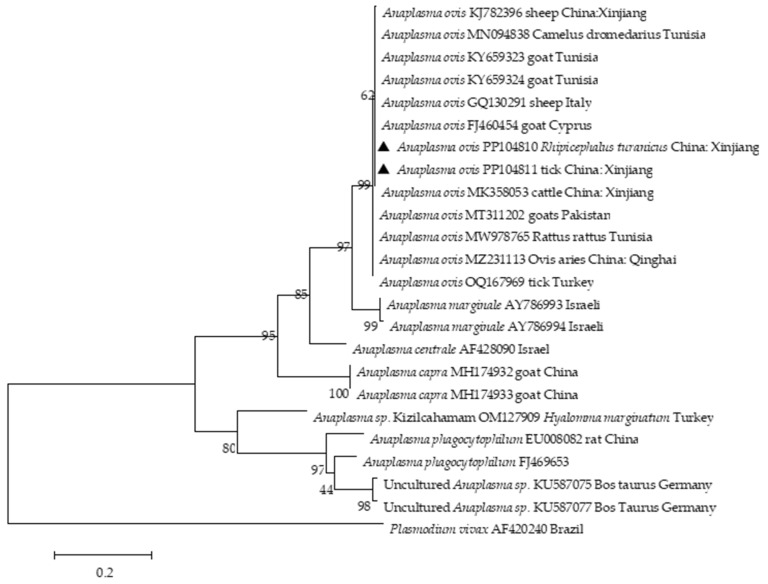
Phylogenetic analysis of *Anaplasma ovis Msp*4 gene sequences was performed using the maximum likelihood method based on the Kimura 2-parameter model. The numbers at the nodes indicate the percentage support derived from 1000 bootstrap replicates. Sequences from this study are denoted by black triangles.

**Figure 4:**
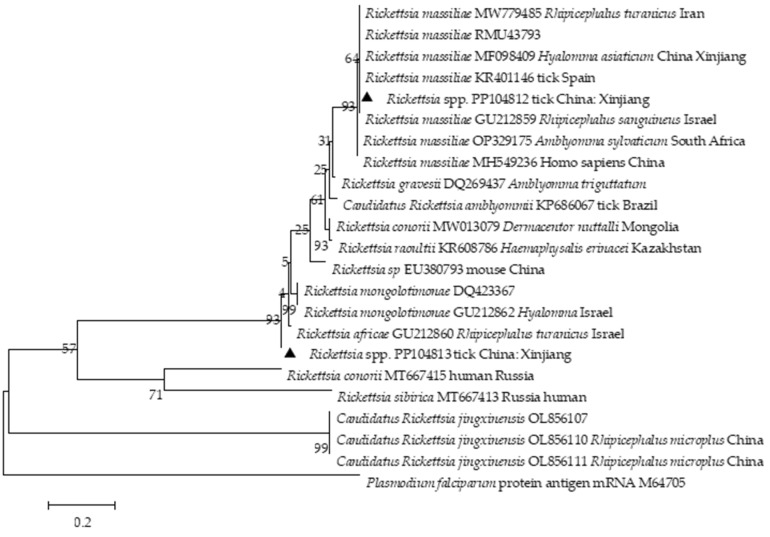
Phylogenetic analysis of *Rickettsia* spp. *OmpA* gene sequences was conducted using the maximum likelihood method based on the Kimura 2-parameter model. The values at the nodes represent the percentage support from 1000 bootstrap replicates. Sequences from this study are indicated by black triangles.

PCR amplicons from positive samples were cloned and sequenced. *T. ovis* 18S rDNA (GenBank Accession no. PP065759, PP065760) from Xinjiang shared a 99.61% sequence identity with *T. ovis* (MN712508). Interestingly, the *T. ovis* 18S rRNA sequences formed a distinct cluster and illustrated a close relationship with the sequences from Iraq (MT732332) and Thailand (OM802548) (Figure 2). Two *A. ovis Msp*4 sequences obtained in this study exhibited a 99.62% identity with *A. ovis* (MN198191). Two isolates (GenBank Accession no. PP104810-PP104811) shared a 99.62% nucleotide sequence identity with *A. ovis* obtained from goats (FJ460454) in Cyprus and sheep (MF002530) in Italy (Figure 3). *Rickettsia* spp. *OmpA* (GenBank Accession no. PP104812) from Xinjiang shared 99.68% and 99.68% sequence identity to *Rickettsia massiliae* (KR401143) and *R. raoultii* (NR043755), respectively. The phylogenetic analysis revealed two distinct *Rickettsia* spp. clades. One clade included the Xinjiang isolate and sequences from Spain (KR401146) and China (Xinjiang, MF098409). The other clade showed a close relationship between PP104813 (*Candidatus Rickettsia barbariae*) and an isolate from Israel (GU212860) (Figure 4).

## 4. Discussion

Xinjiang, a region in China with a complex geographic environment, has experienced a surge in the ruminant trade since the launch of the “Silk Road Economic Belt” initiative. Xinjiang shares extensive borders with eight countries, including India, Pakistan, Afghanistan, Tajikistan, Kyrgyzstan, Kazakhstan, Russia, and Mongolia. Due to this proximity, animal transport across these borders may also increase the likelihood of disease introduction into China. The risk of tick-borne pathogens (TBPs) entering China through animal transport is also increased. Such introductions pose a significant threat to both Xinjiang’s livestock industry and the nation as a whole, as animals carrying disease and tick vectors can be transported throughout China. Despite the importance of understanding TBPs in sheep from southern Xinjiang, knowledge gaps persist regarding their genetic diversity and transmission. This study aimed to identify and characterize the pathogens harbored by two tick species (*Rh. turanicus* and *H. anatolicum*) collected from sheep in southern Xinjiang.

Given the four outbreaks of theileriosis in small ruminants from 2020 to 2022 in other countries, with morbidity rates of 27.95% and mortality rates 17.46% lower than the 62.5% case fatality rate observed in India [13], theileriosis in sheep warrants more attention in Xinjiang. *T. ovis* and *T. lestoquardi* were identified and analyzed in these four outbreaks [13]. The first identified case of *Theileria* in sheep and goats from China dates back to 2007 [14], subsequently identified as *T. ovis*. In the present study, *T. ovis* identified in both *Rh. turanicus* and *H. anatolicum* were found on sheep from southern Xinjiang, China. Additionally, *H. anatolicum* was detected on one of the deceased sheep with a history of contact with the malignant ovine theileriosis outbreak, as reported by Moudgil et al. (2023) [15]. Further investigations in India reported *T. ovis* (29.1%), *T. lestoquardi* (12.69%), and *T. luwenshuni* (5.97%) in sheep [16]. An epidemiological investigation of *T. lestoquardi* was also conducted in Pakistan [3].

The Aksu (Aksu and Wensu) area in the southern part of Xinjiang, near Kazakhstan and Kyrgyzstan, shows the presence of *T. ovis*, which is aligned with findings by Sang (2021) [17] and Sultankulova (2022) [18]. This situation was similar to those in Kizilsu Kirgizil (Akto) and Kashgar (Shufu) regions, near Afghanistan and Pakistan. *T. ovis* has been reported only in Pakistan, where *Rhipicephalus turanicus* has also been identified [4,19]. Studies have also documented the molecular detection of *Theileria* spp. in ticks and ruminants in Kazakhstan, with *Rh. turanicus* identified as a potential vector of *T. ovis* in the southern part of Kazakhstan [17,18]. Our findings support this hypothesis, suggesting *Rh. turanicus* as potential vector for *T. ovis* transmission between Xinjiang, China, and Kazakhstan. Comparable infection rates were observed for *A. marginale* (11%), *A. ovis* (28%), and *T. ovis* (3%) in goats and sheep from Pakistan [4]. Additionally, *Rh. turanicus* and *H. anatolicum* were among over 15 tick species, including *Hyalomma*, *Rhipicephalus*, *Dermacentor*, and *Amblyomma*, identified as carrying these pathogens in Pakistan [20].

To date, limited research has been conducted on *Anaplasma* spp. While economic development has increased in Kazakhstan, Tajikistan, Kyrgyzstan, and Pakistan, studies on *Anaplasma* in these regions remain limited. An overall prevalence of 69.4% (59/85) and 80.5% (70/85) was reported in Mongolia for *A. ovis* in goats and sheep, respectively [21]. *A. ovis* prevalence in goats was 15% (185/1200), including cases reported in Punjab, Pakistan [22]. Although *Rh. turanicus* was not identified in Mongolia, according to Enkhtaivan et al. (2019) [23], *H. asiaticum* was the main vector in Mongolia; therefore, the outcome of their research was similar to that of our study. Our observed *A. ovis* infection rate of 12.5% and the phylogenetic analysis showed resemblance to the sequences found in China [24]. A comparison with studies from India revealed higher *T. ovis* (37.2%) but similar *A. ovis* (15.4%) infection rates in sheep [25]. Moudgil et al. also implicated that *T. lestoquardi*, which causes malignant ovine theileriosis, may also be transmitted by ticks. Moreover, a previous study detected *R. massiliae* and *Candidatus R. barbariae* in *Rh. turanicus* from dogs in Xinjiang, China [8].

Nine *Rickettsia* spp. from the spotted fever group (SFG) were identified, including *R. massiliae* and *Candidatus R. barbariae* [8]. These species were detected in *H. anatolicum* and *Rh. turanicus* [26,27]. Compared to those detected in India, where 29 samples were found to be *R. massiliae* [28]; other Rickettsia species included *Rickettsia conorii*, *Rickettsia asembonensis*, and *Candidatus Rickettsia senegalensis* [28,29]. *Candidatus R. barbariae* has also been identified in ticks from Kazakhstan [30], specifically in *Rh. turanicus*. Hay’s report on Kazakhstan included investigations of *R. sibirica*, *R. conorii*, *R. slovaca*, *R. raoultii*, and *R. aeschlimannii*-like [2]. In previous research, *Candidatus R. barbariae* was found in *Melophagus ovinus* parasitizing the red fox (*Vulpes vulpes*) and *Rh. turanicus* in pet dogs in Xinjiang [8]. *Rh. turanicus* (39.7%) and *H. anatolicum* (11.2%) were identified in equids from Pakistan, while *R. massiliae* was also detected [31]. Shehla et al. (2023) further confirmed *R. massiliae* harbored in *H. anatolicum* and *Rh. turanicus* in Pakistan [32]. Although Kartashov (2020) explored the infection of *Rickettsia* spp. in Tajikistan, no positive cases were reported [33].

## 5. Conclusions

While this study provides valuable insights into the population structures and epidemiological distribution of TBPs in ticks from sheep in southern Xinjiang, China, it did not determine the infection in sheep and is limited by its focus on the tick vector. Future research should investigate the prevalence of the identified pathogens in the blood DNA samples of local ruminants in China and its surroundings to assess the true impact on animal health. Additionally, identifying risk factors for TBDs in sheep at both the individual animal and farm levels is essential for effective disease control and prevention.

In conclusion, *Rh. turanicus* and *H. anatolicum* were the two identified tick species from sheep in southern Xinjiang. *Rickettsia* spp. was the most frequently detected TBP in both species, followed by *Theileria* spp., and *Anaplasma* spp. Co-infections with two or three TBPs were common among tick species in sheep from southern Xinjiang. The sequencing analysis showed that 99–100% of *A. ovis Msp*4 and *Rickettsia* spp. were present in *T. ovis OmpA* from southern Xinjiang. When compared to known isolates from other countries, the *OmpA* sequences displayed 99–100% identity. Our research indicates that *Rickettsia*, *Theileria*, and *Anaplasma* species are prevalent among *H. anatolicum* and *Rh. turanicus* ticks in sheep in southern Xinjiang. This study highlights several important aspects of TBP status in sheep in southern Xinjiang, China, which have serious economic implications for the sheep industry in Asian countries. These aspects should help in the development and implementation of effective prevention and control measures for veterinary practitioners and animal owners.

## Figures and Tables

**Table 1:** The tick-borne pathogens detected in the adult tick pools of *Rh. turanicus* and *H. anatolicum*.

Sample					Number of Positive Tick Pool Pathogen Species (%)		
Tick Species	Area		No. of Sheep	No. of Pool	*A. ovis*	*Rickettsia* spp.	*T. ovis*
***Rh. turanicus***	Aksu	Village A	24	55	5 (9.09)	52 (94.54)	6 (10.91)
		Village B	12	39	2 (5.13)	5 (12.82)	3 (7.69)
		Farm C	12	92	41 (44.57)	65 (70.65)	38 (41.30)
	Wensu	Farm D	20	7	4 (57.14)	7 (100.00)	1 (14.29)
		Farm E	20	9	2 (22.22)	3 (33.33)	5 (55.56)
	Shufu	Village F	20	-	-	-	-
	Akto	Village G	20	6	3 (50.00)	2 (33.33)	2 (33.33)
	Subtotal		128	208	57 (27.40)	134 (64.42)	55 (26.44)
***H. anatolicum***	Aksu	Village A	24	-	-	-	-
		Village B	12	-	-	-	-
		Farm C	12	7	1 (14.29)	1 (14.29)	1 (14.29)
	Wensu	Farm D	20	-	-	-	-
		Farm E	20	57	2 (3.51)	6 (10.53)	19 (33.33)
	Shufu	Village F	20	26	-	1 (3.85)	11 (42.31)
	Akto	Village G	20	-	-	-	-
	Subtotal		128	90	3 (3.33)	8 (8.89)	31 (34.44)
Total			128	298	60 (20.13)	142 (47.65)	86 (28.86)

“-” means not detected; “No.” means the number of ticks tested per animal species.

**Table 2:** Co-infections in *Rh.turanicus* and *H. anatolicum* from southern Xinjiang, China.

Parameter	Hemoparasites	Number of Pools (%, 95%CI)		
		*Rh. turanicus* (N = 208)	*H. anatolicum* (N = 90)	Total (N = 298)
**Single infection**	*A. ovis*	1 (0.48, 0–1.43)	3 (3.33, 0–7.11)	4 (1.34, 0.02–2.66)
	*Rickettsia* spp.	73 (35.10, 28.56–41.64)	4 (4.44, 0.10–8.79)	77 (25.84, 20.84–30.84)
	*T. ovis*	21 (10.10, 5.97–14.22)	27 (30.0, 20.35–39.65)	48 (16.11, 11.91–20.09)
	Sub-total	95 (45.67)	34 (37.78)	129 (43.29)
**Co-infection**	*A. ovis* + *Rickettsia* spp.	32 (15.38, 10.44–20.33)	-	32 (10.74, 7.20–14.27)
	*A. ovis* + *T. ovis*	5 (2.40, 0.31–4.50)	-	5 (1.68, 0.21–3.15)
	*Rickettsia* spp. + *T. ovis*	10 (4.81, 1.88–7.74)	4 (4.44, 0.10–8.79)	14 (4.70, 2.28–7.11)
	*A. ovis* + *Rickettsia* spp. + *T. ovis*	19 (9.14, 5.19–13.08)	-	19 (6.38, 3.59–9.17)

“-” means not detected; “N” means the number of tick pools tested per animal species.

## Data Availability

All data are disclosed in the paper.

## Appendix A

**Table A1:** Sequence information of primers for the molecular biological identification and pathogen detection of ticks.

Name	Gene	Fragment Size	Primers (5′-3′) Forward and Downward	References
Tick species	*CO*I	709 bp	GGTCAACAAATCATA AAGATATTGG	[5]
			TAAACTTCAGGGTGA CCAAAAAATCA	
	16S rDNA	461 bp	TCGTCTGTCTGAGGGTCGGA	
			ATCGTCTCGTGTAGCGTCG	
*Theileria* spp.	18S rRNA	1421 bp	GTCTTGTAATTGGAATGATGG	[12]
			TAGTTTATGGTTAGGACTACG	
*Rickettsia* spp.	OmpA	632 bp	ATGGCGAATATTTCTCCAAAA	[5]
			GTTCCGTTAATGGCAGCATCT	
*Anaplasma* spp.	Msp4	852 bp	GGGAGCTCCTATGAATT ACAGAGAATTGTTTAC	[11]
			CCGGATCCTTAGCTGA ACAGGAATCTTGC	
